# An Assessment of Household and Individual-Level Mosquito Prevention Methods during the Chikungunya Virus Outbreak in the United States Virgin Islands, 2014–2015

**DOI:** 10.4269/ajtmh.17-0799

**Published:** 2018-02-05

**Authors:** Leora R. Feldstein, Ali Rowhani-Rahbar, J. Erin Staples, M. Elizabeth Halloran, Esther M. Ellis

**Affiliations:** 1 Department of Epidemiology, School of Public Health, University of Washington, Seattle, Washington;; 2 Vaccine and Infectious Disease Division, Fred Hutchinson Cancer Research Center, Seattle, Washington;; 3 Division of Vector-Borne Diseases, National Center for Emerging and Zoonotic Infectious Diseases, Centers for Disease Control and Prevention, Fort Collins, Colorado;; 4 Center for Inference and Dynamics of Infectious Diseases, Fred Hutchinson Cancer Research Center, Seattle, Washington;; 5 Department of Biostatistics, School of Public Health, University of Washington, Seattle, Washington;; 6 United States Virgin Islands Department of Health, Saint Croix, United States Virgin Islands

## Abstract

Recent large-scale chikungunya virus (CHIKV) and Zika virus epidemics in the Americas pose a growing public health threat. Given that mosquito bite prevention and vector control are the main prevention methods available to reduce transmission of these viruses, we assessed adherence to these methods in the United States Virgin Islands (USVI). We interviewed 334 USVI residents between December 2014 and February 2015 to measure differences in mosquito prevention practices by gender, income, presence of CHIKV symptoms, and age. Only 27% (91/334) of participants reported having an air conditioner, and of the 91 with air-conditioners, 18 (20%) reported never using it. Annual household income > $50,000 was associated with owning and using an air conditioner (41%; 95% confidence interval [CI]: 28–53% compared with annual household income ≤ $50,000: 17%; 95% CI: 12–22%). The majority of participants reported the presence of vegetation in their yard or near their home (79%; 265) and a cistern on their property (78%; 259). Only 52 (16%) participants reported wearing mosquito repellent more than once per week. Although the majority (80%; 268) of participants reported having screens on all of their windows and doors, most (82%; 273) of those interviewed still reported seeing mosquitoes in their homes. Given the uniformly low adherence to individual- and household-level mosquito bite prevention measures in the USVI, these findings emphasize the need for improved public health messaging and investment in therapeutic and vaccine research to mitigate vector-borne disease outbreaks.

## INTRODUCTION

Mosquitoes and the infectious diseases they transmit continue to be a significant public health challenge globally. Recent large-scale chikungunya virus (CHIKV) and Zika virus (ZIKV) epidemics in the Americas and their associated severe outcomes illustrate this risk.^1,2^ Furthermore, no antiviral or therapeutic treatment or vaccine presently exists for these diseases.^1,3^ Presently, mosquito bite prevention and vector control are the main prevention methods available to reduce the transmission of CHIKV, ZIKV, and dengue virus (DENV).^4^

The United States Virgin Islands (USVI) was one of the many regions in the Caribbean affected by the CHIKV epidemic in 2014.^5^ In response to the initial cases of CHIKV, the USVI Department of Health (DOH) worked in collaboration with the Centers for Disease Control and Prevention to educate health-care providers and the public regarding CHIKV disease and to provide recommendations for individual- and household-level mosquito prevention in the form of media campaigns and educational materials.

To assess adherence to these individual- and household-level mosquito prevention methods and to identify groups where additional targeted prevention messaging might be warranted, we interviewed 334 USVI residents between December 2014 and February 2015. Differences in mosquito prevention practices were evaluated by gender, income, presence of CHIKV symptoms, and age.

## METHODS

Participants were identified from two difference sources. First, we invited USVI residents who had symptoms compatible with CHIKV infections, specifically fever and polyarthralgia, and tested positive for evidence of a recent CHIKV infection from June 2014 to February 2015 to participate. Second, we invited USVI residents who sought health care from December 2014 to February 2015, and their accompanying family members to participate in the study (Supplemental Appendix). The second group, nonsymptomatic controls, consisted of residents of any age in the waiting room of the emergency room of a hospital or at a health clinic in the USVI. These individuals were either waiting to be seen by a clinician or were accompanying a relative or friend who was waiting to be seen. The individuals were asked about symptoms compatible with CHIKV infection since June 2014 and were excluded if they reported having fever and polyarthralgia, to compare whether symptoms influenced behavior (Supplemental Appendix).

The interviews were conducted from December 2014 to February 2015, and individuals were asked about the use of mosquito repellent and air conditioners, emptying of water containers, household characteristics, and the amount of time spent outdoors per day.

Descriptive statistics were used to summarize and compare frequencies of household characteristics and individual-level practices of vector control by gender, income, symptoms of acute CHIKV infection, and age. The χ^2^ and Fisher’s exact tests were used to test for statistical significance. Data were analyzed using STATA14.0™ (StataCorp 2015, College Station, TX).

### Ethics statement.

Verbal informed consent was obtained from all participants before interviewing them. Parental/guardian consent was acquired on behalf of all child participants and parents/guardians responded for children under the age of 12. Verbal informed consent was documented on the questionnaire by the interviewer and entered into the database. Oral consent was used because of the fact that almost half of the interviews took place over the phone. Ethics approval for this study, as well as the use of verbal consent was obtained from the University of the Virgin Islands and the University of Washington.

## RESULTS

Three hundred and thirty-four USVI residents were interviewed from December 2014 to February 2015. Of all participants, 220 (66%) were female and the median age was 42 years (interquartile range of 23–58 years). A total of 269 (81%) participants provided information on their household income; of whom, 78% (210) reported making ≤ $50,000/year. Almost half (155; 46%) of all participants reported symptoms consistent with CHIKV infection and tested positive for evidence of recent infection. The remainder denied having any symptoms of CHIKV infection since June 2014 when the virus was first recognized in the USVI.

Of the 334 participants, the majority (268; 80%) reported having screens on all of their windows and doors, but very few (24; 7%) reported having screens on their porch or patio and most (273; 82%) reported seeing mosquitoes in their homes (Table 1). Ninety-one (27%) residents reported having an air conditioner (an effective tool for keeping mosquitoes out of homes) but 18 (20%) reported never using it and 32 (35%) reported using the air conditioner only at night. The majority (265; 79%) of individuals reported the presence of vegetation (trees or plants) in their yard or near their home and had a cistern (259; 78%) on their property. Almost all participants (298; 89%) reported either not owning uncovered containers or emptying uncovered containers at least once a week. One out of every four participants (*N* = 86; 26%) reported spending more than 5 hours on average outside per day. However, only 16% (*N* = 52) of all participants reported wearing mosquito repellent more than once per week.

**Table 1:** Differences in household characteristics and individual-level mosquito bite prevention measures by gender, income, presence of chikungunya symptoms, and age

	All individuals (*N* = 334)		Gender				Income				Chikungunya virus symptoms				Age (years)					
			Males (*N* = 114)		Females (*N* = 220)		≤ $50,000 (*N* = 210)		> $50,000 (*N* = 59)		Yes (*N* = 155)		No (*N* = 179)		≤ 35 (*N* = 135)		36–55 (*N* = 102)		> 55 (*N* = 97)	
	*n*	(%)	*n*	(%)	*n*	(%)	*n*	(%)	*n*	(%)	*n*	(%)	*n*	(%)	*n*	(%)	*n*	(%)	*n*	(%)
Screens on all windows	268	(80)	94	(82)	174	(79)	162	(77)	48	(81)	130	(84)	138	(77)	107	(79)	82	(80)	79	(81)
Screen on porch/patio	24	(7)	13	(11)	11	(5)	12	(6)	4	(7)	12	(8)	12	(7)	11	(8)	6	(6)	7	(7)
Has an air conditioner unit	91	(27)	34	(30)	57	(26)	**45**	**(21)**	**29**	**(49)**	55	(35)	36	(20)	32	(24)	36	(35)	23	(24)
Air conditioner use (*N* = 91)																				
All of the time	13	(14)	2	(6)	11	(19)	6	(13)	2	(7)	10	(18)	3	(8)	5	(16)	6	(17)	2	(9)
Only at night	32	(35)	13	(38)	19	(33)	16	(36)	12	(43)	14	(25)	18	(50)	14	(44)	12	(33)	6	(26)
Only in the summer	27	(30)	11	(32)	16	(28)	14	(31)	10	(36)	17	(31)	10	(28)	8	(25)	10	(28)	9	(39)
Never	18	(20)	8	(24)	10	(18)	9	(20)	4	(14)	14	(25)	4	(11)	5	(16)	7	(19)	6	(26)
Unknown	1	(1)	0	(0)	1	(2)	0	(0)	1	(3)	0	(0)	1	(3)	0	(0)	1	(3)	0	(0)
Sees mosquitoes in house	273	(82)	92	(81)	181	(82)	167	(80)	50	(85)	133	(86)	140	(78)	110	(81)	82	(80)	81	(84)
Uncovered water containers																				
Does not own any	175	(52)	56	(49)	119	(54)	112	(53)	28	(47)	85	(55)	90	(50)	73	(54)	55	(54)	47	(48)
Empties ≥ 1 week	123	(37)	41	(36)	82	(37)	77	(37)	24	(41)	52	(34)	71	(40)	48	(36)	36	(35)	39	(40)
Do not know/unknown	8	(2)	3	(3)	5	(2)	5	(2)	0	(0)	4	(3)	4	(2)	4	(3)	1	(1)	3	(3)
Property contains																				
Potted plants	165	(49)	59	(52)	106	(48)	**91**	**(43)**	**40**	**(68)**	86	(55)	79	(44)	58	(43)	50	(49)	57	(59)
Vegetation	265	(79)	95	(83)	170	(77)	162	(77)	54	(92)	126	(81)	139	(78)	101	(75)	87	(85)	77	(79)
Buckets	112	(34)	**51**	**(45)**	**61**	**(28)**	72	(34)	21	(36)	48	(31)	64	(36)	49	(36)	31	(30)	32	(33)
Pool	20	(6)	6	(5)	14	(6)	**8**	**(4)**	**11**	**(19)**	10	(6)	10	(6)	5	(4)	6	(6)	9	(9)
Boat	4	(1)	2	(2)	2	(1)	3	(1)	0	(0)	2	(1)	2	(1)	2	(1)	0	(0)	2	(2)
Used car tires	21	(6)	10	(9)	11	(5)	15	(7)	3	(5)	7	(5)	14	(8)	7	(5)	7	(7)	7	(7)
Cistern	259	(78)	94	(82)	165	(75)	162	(77)	53	(90)	123	(79)	136	(76)	100	(74)	81	(79)	78	(80)
Outside > 5 hours per day	86	(26)	**45**	**(39)**	**41**	**(19)**	57	(27)	19	(32)	27	(17)	59	(33)	40	(30)	21	(21)	25	(26)
Wears repellent > 1 per week	52	(16)	16	(14)	36	(16)	29	(14)	8	(14)	**33**	**(21)**	**19**	**(11)**	20	(15)	18	(18)	14	(14)

Statistically significant differences (*P* < 0.05) are highlighted in bold.

When different mosquito bite exposures or prevention measures were compared by gender, males were significantly more likely to spend more than 5 hours per day outdoors than females (39%; 95% confidence interval [CI]: 31–49% and 19%; 95% CI: 14–25%, respectively). A greater proportion of males also reported having more buckets in their yard or near their home than females (45%; 95% CI: 36–54% and 28%; 95% CI: 22–34%, respectively, *P* < 0.01). There were no statistically significant differences by gender for all other characteristics, including having window screens and screened-in porches, owning an air conditioner unit, wearing mosquito repellent, and having different types of water-holding containers.

We identified three different mosquito prevention factors that varied based on annual household income. Individuals reporting an annual household income of > $50,000 were significantly more likely to own and use an air conditioner than those who reported an annual household income ≤ $50,000 (41%; 95% CI: 28–53% and 17%; 95% CI: 12–22%, respectively). A significantly greater proportion of individuals with an annual household income > $50,000 reported having potted plants in and near their homes and having swimming pools compared with those with a household income ≤ $50,000 (potted plants: 68%; 95% CI: 54–79% and 43%; 95% CI: 37–50%, respectively, *P* < 0.01 and pools: 19%; 95% CI: 9–29% and 4%; 95% CI: 1–7%, *P* < 0.01). No other household characteristics or individual behavior practices varied significantly by income.

A greater proportion of participants with symptoms of CHIKV infection reported wearing mosquito repellent more than once per week than nonsymptomatic participants (21%; 95% CI: 15–29% and 11%; 95% CI: 7–16%, respectively, *P* < 0.01). No other household characteristics or individual behavior practices varied significantly by the presence of CHIKV symptoms in the 6–7 months before the interview.

Participants aged > 55 years tended to use their air conditioners less than the younger participants, and a greater proportion of them owned potted plants but none of these characteristics or any others varied significantly by age group (Table 1).

## DISCUSSION

These results indicate that the proportion of persons surveyed in the USVI who practiced mosquito bite prevention measures was low and generally not affected by gender, income, symptoms of CHIKV infection, or age. This might be due to the general attitudes and life styles of the population, the tropical climate, and challenges in promoting behavior change.

Twenty-three percent of USVI residents live below the poverty line^6^; thus, for much of the USVI population, owning an air conditioner and being able to afford the electricity to use it may be unfeasible. This is consistent with our findings that lower income households were significantly less likely to own an air conditioner. Air conditioners are extremely effective at keeping mosquitoes out of homes because mosquitoes avoid cool environments, but the use and upkeep of air conditioning units are expensive.

Home improvements such as screening in porches and altering landscapes to avoid standing water, the most common breeding site for *Aedes* spp. mosquitoes,^7^ may be too costly. Moreover, because of the humid climate and considerable rainfall in the USVI (1,023 mm per year on average), the presence of standing water is inevitable.^8^ Even if residents are dutiful about emptying their own containers of water, as was found in this study, or picking up trash, *Aedes* spp. mosquito can travel up to 230 m and hence could come from other sources beyond the person’s yard.^9,10^ In this scenario, neighborhood-wide sanitation activities may be more effective at reducing mosquito-breeding grounds.

More than three-quarters of residents reported having water cisterns. Cisterns are vital for storing rainwater but are also attractive breeding sites for mosquitoes.^11,12^ This situation is not unique to the USVI. The percent of people living below the poverty line in Latin American and Caribbean countries with the ongoing ZIKV transmission ranges from 21% to 70%.^13^ Many of these countries experience even more rainfall than the USVI and have similar rain storage practices.^14,15^

The low percentage of individuals who reported using mosquito repellent on a regular basis may indicate that media campaigns, educational materials, and even distributing free mosquito repellent by the USVI DOH during the CHIKV outbreak may not be adequate to promote behavior change. The only factor that was significantly associated with increased use of mosquito repellent was having symptoms with CHIKV disease. It is possible that this difference is present because participants with CHIKV symptoms were told by their health-care providers to use repellent and may apply repellent more often because they do not want to suffer from another vector-borne disease. It is also possible that this finding is due to recall bias; knowing that mosquito repellent is an effective measure against CHIKV, participants with CHIKV symptoms may have been more likely to report repellent use than nonsymptomatic individuals. Perhaps, using human interest stories in campaigns or recruiting well-known community members who became sick from the disease could improve the effectiveness of messaging. However, even if persons do adhere to individual mosquito bite avoidance measures it remains unclear how effective they are at preventing mosquito-borne diseases.

The present study was subject to certain limitations. We included only individuals who sought health care and their accompanying family members. Therefore, these results may not be representative of the larger USVI population, particularly those who might not have health insurance that could limit their access to care. Most of the nonsymptomatic participants were not tested for evidence of CHIKV infection. As a result, we cannot draw any conclusions about whether any of these characteristics are associated with the risk for CHIKV infection. Because of the exploratory nature of this survey, we did not assess potential confounding factors for individual prevention measures. Finally, we did not ask questions specifically about exposure to prevention messaging. Therefore, we cannot assume that the failure to use mosquito bite prevention measures was due to ineffective or inadequate prevention messaging.

The results from this study and from prior studies highlight the need for large-scale prospective intervention studies to more accurately assess the effectiveness of individual mosquito bite prevention measures and associated public health messages. Personal protective measures by an individual or a family may not reduce the mosquito population enough to halt disease transmission. Instead, vector control may only be sufficient at the community- or population level. This report continues to highlight the challenges in having good uptake on personal prevention measures to prevent mosquito-borne diseases. Given these challenges, it is important to continue to explore effective ways to prevent these diseases including alternative community-level vector control strategies (e.g., Wolbachia) and development of vaccines. Finally, this study emphasizes the continued need for investment in therapeutic and vaccine research to mitigate the ongoing ZIKV epidemic and prevent future CHIKV and DENV outbreaks.

## Supplementary Material

Supplemental Appendix
