# Supplementary material for: An Assessment of Household and Individual-Level Mosquito Prevention Methods during the Chikungunya Virus Outbreak in the United States Virgin Islands, 2014–2015
Source: Am J Trop Med Hyg. 2018 Feb 5;98(3):845–8. doi: 10.4269/ajtmh.17-0799 (PMC5930869; doi:10.4269/ajtmh.17-0799)
Supplement: Supplementary file 1 [file tpmd170799.SD1.pdf]

SUPPLEMENTAL APPENDIX

Supplemental Table A1

Enrollment of chikungunya virus (CHIKV) cases from December 2014 to February 2016 over the phone

Enrollment process	Number enrolled
Laboratory-confirmed CHIKV cases	457
Missing phone number	116
Incorrect phone number or not in service	93
Did not answer the phone after three attempts	70
Hung up before the questionnaire was completed	10
Refused to participate	11
Died	2
Total enrolled	155

Supplemental Table A2

Number of nonsymptomatic controls interviewed in-person

Total number interviewed	179
